# NF-κB2 signalling in enteroids modulates enterocyte responses to secreted factors from bone marrow-derived dendritic cells

**DOI:** 10.1038/s41419-019-2129-5

**Published:** 2019-11-26

**Authors:** Lauren G. Jones, Andra Vaida, Louise M. Thompson, Felix I. Ikuomola, Jorge H. Caamaño, Michael D. Burkitt, Fabio Miyajima, Jonathan M. Williams, Barry J. Campbell, D. Mark Pritchard, Carrie A. Duckworth

**Affiliations:** 1 Gastroenterology Research Unit, Department of Cellular and Molecular Physiology, Institute of Translational Medicine, University of Liverpool, Liverpool, UK; 2 Institute of Immunology and Immunotherapy, College of Medical and Dental Sciences, University of Birmingham, Birmingham, UK; 3 Department of Clinical and Molecular Pharmacology, Institute of Translational Medicine, University of Liverpool, Liverpool, UK; 4 Biotechnology Group, Oswaldo Cruz Foundation, Ceara, Brazil; 5 Pathobiology and Population Sciences, Royal Veterinary College, Hawkshead Lane, North Mymms, Hatfield, Hertfordshire UK

**Keywords:** Intestinal stem cells, Inflammatory bowel disease, Experimental models of disease, Immunopathogenesis, Sepsis

## Abstract

Alternative pathway NF-κB signalling regulates susceptibility towards developing inflammatory bowel disease (IBD), colitis-associated cancer and sepsis-associated intestinal epithelial cell apoptosis and shedding. However, the cell populations responsible for the perturbed alternative pathway NF-κB signalling in intestinal mucosal pathology remain unclear. In order to investigate the contribution of the epithelial compartment, we have tested whether NF-κB2 regulated transcription in intestinal epithelial cells controls the intestinal epithelial response to cytokines that are known to disrupt intestinal barrier permeability. Enteroids were generated from the proximal, middle and distal regions of small intestine (SI) from C57BL/6J wild-type mice and displayed region-specific morphology that was maintained during sub-culture. Enteroids treated with 100 ng/mL TNF were compared with corresponding regions of SI from C57BL/6J mice treated systemically with 0.33 mg/kg TNF for 1.5 h. TNF-induced apoptosis in all regions of the intestine in vitro and in vivo but resulted in Paneth cell degranulation only in proximal tissue-derived SI and enteroids. TNF also resulted in increased enteroid sphericity (quantified as circularity from two-dimensional bright field images). This response was dose and time-dependent and correlated with active caspase-3 immunopositivity. Proximal tissue-derived enteroids generated from *Nfκb2*^−*/−*^ mice showed a significantly blunted circularity response following the addition of TNF, IFNγ, lipopolysaccharide (LPS) activated C57BL/6J-derived bone marrow-derived dendritic cells (BMDC) and secreted factors from LPS-activated BMDCs. However, *Nfκb1*^*−/−*^ mouse-derived enteroids showed no significant changes in response to these stimuli. In conclusion, the selection of SI region is important when designing enteroid studies as region-specific identity and response to stimuli such as TNF are maintained in culture. Intestinal epithelial cells are at least partially responsible for regulating their own fate by modulating NF-κB2 signalling in response to stimuli known to be involved in multiple intestinal and systemic diseases. Future studies are warranted to investigate the therapeutic potential of intestinal epithelial NF-κB2 inhibition.

## Introduction

The intestine is lined by a single layer of columnar epithelial cells that function to maintain a barrier between luminal contents and the body^1,2^. Barrier function is maintained by the tight regulation of intestinal epithelial cell proliferation, apoptosis and cell shedding rates which are themselves modulated by several factors, including age, genetic background, dietary components, intestinal microbiota, enterally and parenterally administered drugs and other environmental factors^3^. Dysregulation of these cellular processes results in the break-down of the intestinal barrier and this is thought to be a contributing factor to the development of multiple diseases. We have recently shown that defective intestinal barrier function caused by cell shedding in the terminal ileum predicts relapse of inflammatory bowel disease (IBD) in humans^4^. Defects in intestinal barrier function may also be involved in the pathogenesis of systemic conditions, such as metabolic endotoxaemia and sepsis^5,6^.

Several pro-inflammatory cytokines are present at increased concentrations in the circulation, intestinal lumen and lamina propria of the intestine during active IBD, infectious diarrhoea, coeliac disease and sepsis^7–12^. Tumour necrosis factor (TNF) is a well characterised cytokine that is produced in these conditions and is a key mediator of mucosal pathology^3,13^. Elevated TNF concentrations in excess of 63 ng/g stool have been documented in humans in response to *Shigella flexneri* infection^11^. Anti-TNF therapies are also widely used clinically to ameliorate active Crohn’s disease^14^.

We have recently shown that the administration of lipopolysaccharide (LPS) or its downstream effector, TNF, by intraperitoneal injection to mice results in a massive induction of epithelial apoptosis and cell shedding from the SI villus tip within 1.5 h^4,15–17^. This rapid onset of active caspase-3 positively stained shedding cells subsequently resulted in villus atrophy and was accompanied by fluid effusion into the SI lumen and diarrhoea, but was largely diminished at 3 h post LPS injection^17^. However, increased efflux of FITC-dextran (FD4) from the intestinal lumen into the circulation following LPS treatment was observed at later time-points^17^, suggesting that defects in intestinal barrier function persist once cell shedding and apoptosis have subsided, until complete restitution of the epithelium has been achieved. The regenerative capacity of the intestinal epithelium is remarkable. Cell turnover in the epithelium is normally around 5 days with around 1400 cells shed from a single mouse villus tip per day^18^. We would therefore anticipate that barrier function could take up to 5 days to be restored following extensive epithelial cell loss by apoptosis and cell shedding once inflammatory stimuli such as TNF and interferon γ (IFNγ) have been removed. Understanding the mechanisms underpinning intestinal epithelial cell protection from cytokine-mediated injury will enable the future development of therapeutics for several intestinal and systemic diseases.

The NF-κB family of transcription factors consists of 5 members (NF-κB2 (p52), RelB, NF-κB1 (p50), c-Rel, and RelA (p65)) and regulate multiple cellular processes^19^. We have recently identified components of the alternative NF-κB signalling pathway that are important in modulating the susceptibility to IBD, colitis-associated cancer and intestinal epithelial apoptosis and cell shedding in mice. *Nfκb2*^*−/−*^ mice were resistant to dextran sulphate sodium (DSS)-induced colitis and azoxymethane/DSS-induced colonic adenoma formation^20^ and were also resistant to the induction of LPS and TNF-induced SI apoptosis and cell shedding in vivo^16,17^. Infection studies have also shown that *Nfκb2*^*−/*−^ mice have a reduced ability to clear the gut helminth *Trichuris muris*^21^ and the gastric pathogen *Helicobacter felis*, the latter observation being associated with reduced gastric preneoplastic pathology owing to a defective immune response^22^. The complex in vivo nature of these studies to date, has made it impossible to determine the importance of each cellular compartment (e.g., immune or epithelial) in regulating the intestinal response to damage-inducing stimuli. The majority of studies characterising alternative pathway NF-κB signalling are also focused on immune cell mechanisms which may be differentially regulated in other cellular compartments. Therefore, we have now generated a SI enteroid model to assess the interactions between the intestinal epithelium and specific immune cell derived cytokines that are known to be elevated in intestinal and systemic disease, and to determine the importance of epithelial cell-specific alternative pathway NF-κB signalling in regulating the epithelial cell response to cytokine-induced pathology.

Dendritic cells are present throughout the lamina propria of the gut and contribute to innate and adaptive immunity thus regulating gut homoeostasis. Direct bidirectional interaction of dendritic cells with the epithelium is believed to maintain the dendritic cell population in a tolerogenic state and this protects the intestinal epithelium from inappropriate immune cell-derived attack^23^. Whilst dendritic cells are the most potent antigen presenting cell population, they are also activated by pro-inflammatory cytokines or oxidative stress and are conditioned by their surrounding microenvironment^24^. Cytokines and chemokines released by dendritic cells along with other lamina propria immune cell populations can modulate intestinal barrier integrity, intestinal cell proliferation and cell death^25^. Our previous whole mouse global *Nfκb2* knockout studies^17,20,22^ have limited our ability to dissect the importance of alternative pathway NF-κB signalling within epithelial and immune compartments in regulating the susceptibility of the intestinal epithelium to cytokine-induced injury. We therefore generated a bone marrow-derived dendritic cell (BMDC) reconstituted intestinal organoid model to assess the role of NF-κB2 in regulating intestinal epithelial cell-specific responses to secreted factors from BMDCs.

We hypothesise that *Nfκb2* activation within intestinal epithelial cells sensitises them to the induction of apoptosis by pro-inflammatory cytokines that are upregulated in intestinal tissues and systemically during active intestinal disease and bacteraemia. We have therefore explored whether Nfκb2 inhibition within intestinal epithelia could be a potential therapeutic approach to ameliorating inflammation-associated intestinal disease using a novel reconstituted intestinal organoid co-culture model.

## Results

### Regional differences are observed in enteroid morphology

**Fig. 1:**
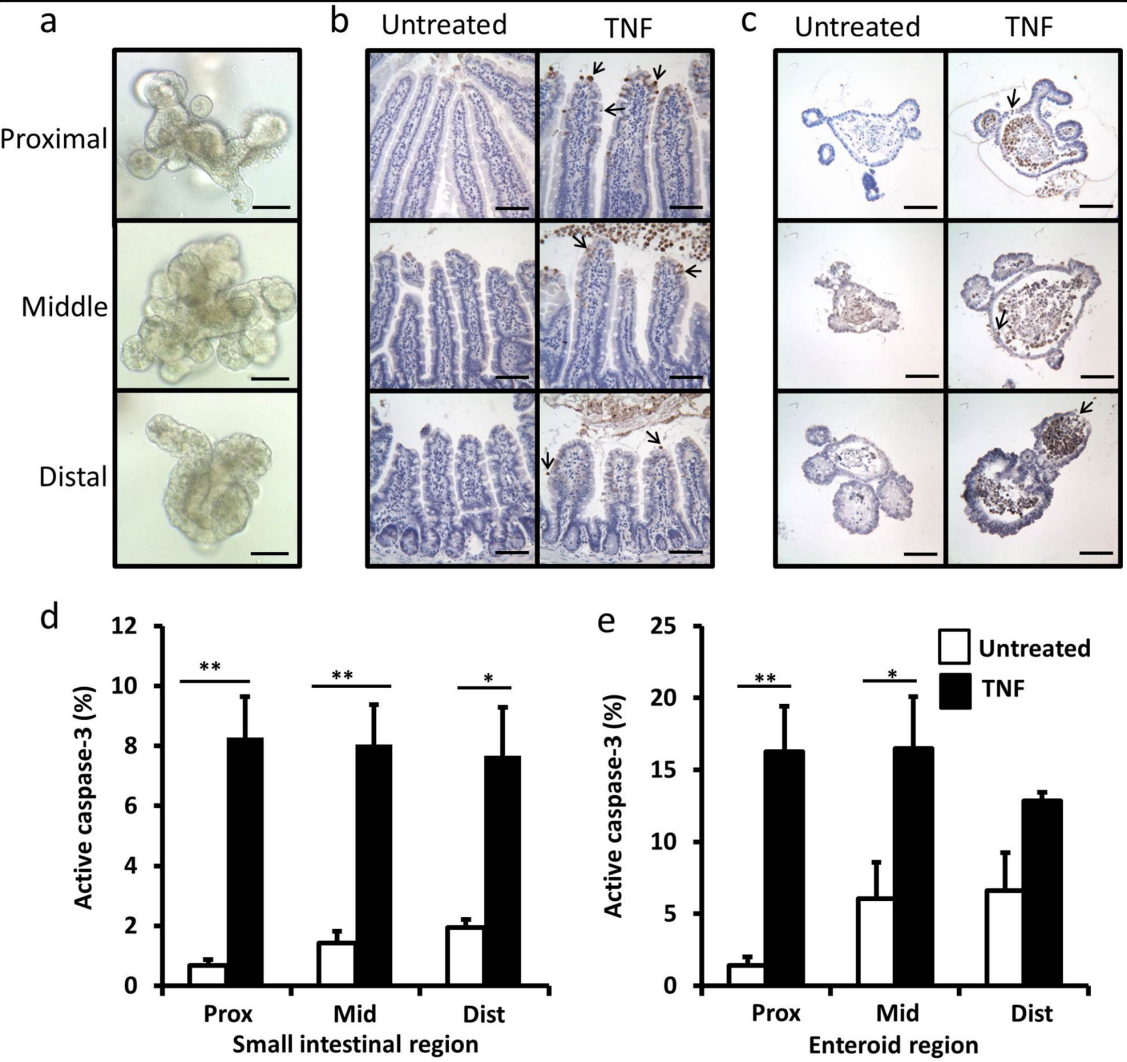
Enteroids show regional differences in morphology and respond to TNF similarly to native SI tissue. Bright field micrographs of enteroids derived from proximal, middle and distal sections of C57BL/6J SI (**a**). Proximal, middle and distal sections of SI tissue taken from untreated and TNF (0.33 mg/kg, 1.5 h) treated C57BL/6J mice (**b**) and enteroids generated from proximal, middle and distal regions of SI untreated or treated with TNF (100 ng/mL 24 h) (**c**) immunohistochemically stained for active caspase-3. Quantification of active caspase-3 positive epithelial cells from untreated (white) and TNF treated (black) SI epithelia (**d**) and enteroid culture (**e**). For animal study, quantified from *n* = 6 mice per group; for enteroid study *n* = 6, *N* = 3, *p < 0.05, **p < 0.01. Scale bars = 100 µm.

The small intestine (SI) displays regional differences in structure and function. The expression of several genes is altered along the cephalocaudal axis in vivo and this region-specific identity has previously been shown to be maintained in enteroid culture^26,27^. We therefore generated organoids from proximal, middle and distal 2 cm segments of murine SI from female C57BL/6J mice and assessed their morphological changes over 10 consecutive passages. Enteroids derived from the three distinct areas displayed morphological differences between regions and these did not alter following repeated passage. At days 3–5 post passage, proximal SI derived enteroids had the longest (116 ± 3 μm) and most abundant crypt domains (7.22 ± 0.26/enteroid) with smooth surfaces and a polarised Paneth cell distribution at the base of crypts, whilst the distal SI derived enteroids had a smaller number of crypt domains (4.04 ± 1.02/enteroid) which were shorter (89 ± 4 μm) and displayed a more rounded and rugged appearance with a less polarised Paneth cell distribution with these differentiated cells also being found further along the crypt axes (Fig. 1a). Middle SI derived enteroids had 5.22 ± 1.90 crypts per enteroid that were 91 ± 11 μm in length and showed the greatest degree of variation in crypt length and number and Paneth cell distribution, suggestive of an intermediate phenotype.

### TNF induces enteroid apoptosis and Paneth cell depletion, but does not modulate enteroid proliferation

We previously identified subtle regional differences in the murine SI in vivo in response to intraperitoneally administered LPS. A greater extent of villus blunting was observed in the proximal SI compared with other SI regions^17^. We therefore investigated the apoptotic and proliferative responses to a downstream effector of LPS, TNF, in region-specific enteroid cultures compared to histological sections taken from the same regions of the intestine in vivo. Baseline amounts of apoptosis in both untreated SI and in enteroid culture regardless of region were low, however, a small increase in the percentage of active caspase-3 positive cells was observed along the cephalocaudal axis in both systems (Fig. 1b–e). As expected, intraperitoneal injection of 0.33 mg/kg TNF-induced a significant increase in apoptotic cells at the villus tip after 1.5 h (Fig. 1b, d), whilst 100 ng/ml TNF-induced apoptosis to a similar extent in enteroids after 24 h (Fig. 1c, e).

**Fig. 2:**
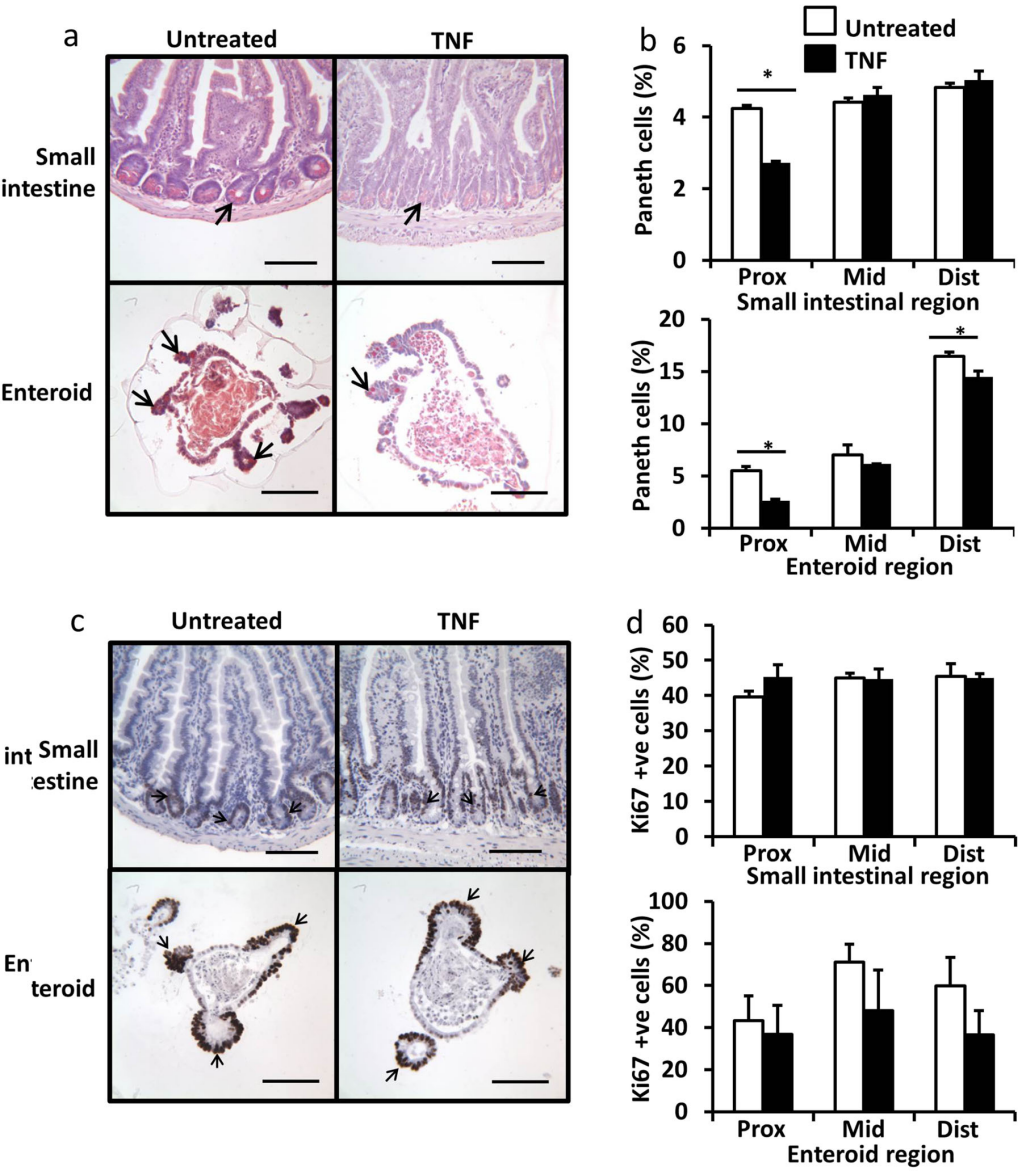
TNF causes Paneth cell degranulation in proximal SI but does not change the rate of cell proliferation. Proximal SI and enteroids stained with Sirius red (**a**). Percentage of Sirius red stained Paneth cells in untreated (white) and TNF treated (black) proximal, middle and distal derived SI (top) and enteroids (bottom) (**b**). Proximal SI and enteroids stained with Ki67 (**c**). Percentage of Ki67 stained epithelial cells in untreated (white) and TNF treated (black) proximal, middle and distal derived SI (top) and enteroids (bottom) (**d**). For animal study quantified from *n* = 6 mice per group, for enteroid study *n* = 6, *N* = 3, *p < 0.05. Scale bars = 100 µm.

Paneth cells are important for maintaining the intestinal stem cell niche, although the exact mechanisms by which this cell population supports the proliferative capacity and number of neighbouring crypt base columnar cells remain unknown. However, TNF has previously been shown to induce Paneth cell dysfunction in vivo^28^, and this could potentially impact on the regenerative capacity of the intestinal epithelium. We therefore investigated whether TNF modulated Paneth cell number in SI tissue and in enteroids as this could impact on crypt-villus growth dynamics. TNF administration caused a more marked and significant reduction in Paneth cell number in the proximal SI and proximal tissue-derived enteroids compared with other SI regions (Fig. 2a, b) suggesting that TNF may modulate the intestinal stem cell niche in different ways within the proximal SI compared with other intestinal regions. Olfactomedin-4 (Olfm4) is a putative active stem cell marker that also labels early transit-amplifying cells. Olfm4 expression was not altered in SI tissues treated with TNF but due to the distribution differences in cellular location of Olfm4 observed in enteroids, Olfm4 expressing cells were not possible to quantify reliably (Supplementary Fig. 1). We also wanted to test whether another abundant secretory cell type was affected by the addition of TNF and found that the goblet cell population in different regions of SI and in enteroids was not modulated by the addition of TNF (Supplementary Fig. 2).

Proliferation in the SI in vivo occurs within the transit-amplifying region of the crypt; a distinctly different part of the crypt-villus axis to the villus tip, which is the region most susceptible to apoptosis in response to systemically administered TNF. As we have shown that TNF causes apoptosis and Paneth cell depletion at the dose and time-points tested in mice in vivo and in enteroids, we wanted to determine whether this treatment had any impact on intestinal proliferation. Proliferation indicated by Ki67 positively stained nuclei was identified in the crypt domains of whole SI tissue and enteroids. TNF treatment had no significant effect on cellular proliferation either in vivo or in enteroid culture, in any of the three SI regions at the doses and time points that showed an increased apoptotic response and Paneth cell depletion (Fig. 2c, d).

### Apoptosis and resultant cell shedding into the lumen correlate positively with enteroid circularity

**Fig. 3:**
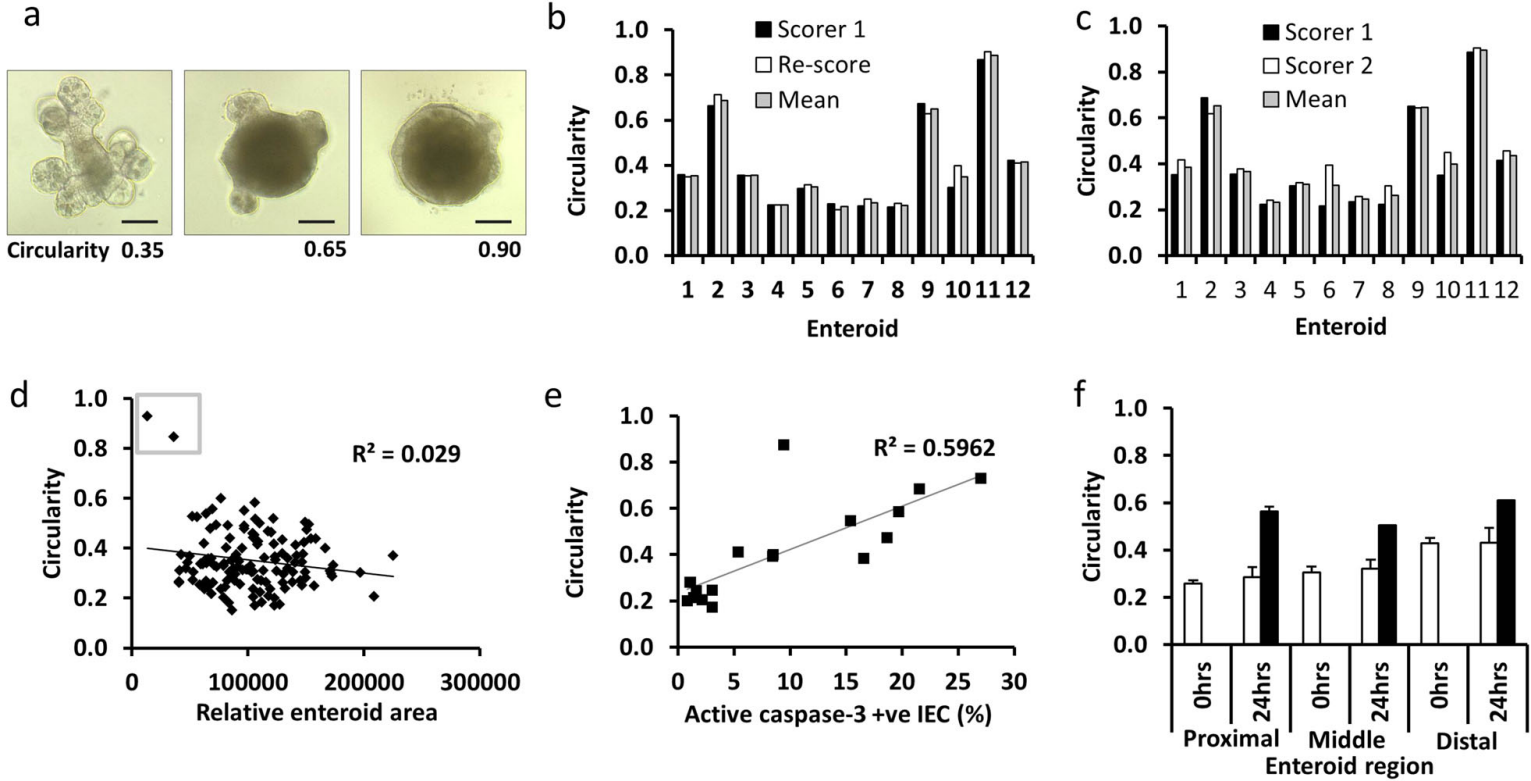
Validation of circularity assessment as a marker of TNF-induced apoptosis in enteroid culture. Light micrograph examples of proximal tissue-derived enteroids either untreated 3 days post passage (left), 48 h following 100 ng/ml TNF (middle) and 48 h following treatment with CM from LPS-activated BMDCs (right) traced in yellow using Image J (**a**). Quantification of circularity from 12 different enteroids administered with a range of treatments over different experiments and re-scored by the same observer (**b**) and by an independent second observer (**c**). Correlation between relative enteroid area and circularity for 130 individual enteroids at baseline (**d**), small organoids at baseline have high circularity values and need to be excluded from further analysis (grey box). Percentage active-caspase 3 positive cells were correlated with enteroid circularity from organoids selected at 0, 24 and 48 h post treatment with 100 ng/ml TNF (**e**). Change in circularity between three enteroid regions at baseline (white bars) and 24 h after 100 ng/ml TNF (black bars). Scale bars = 100 µm.

Villus shortening occurs in vivo as a result of an increased rate of apoptosis and cell shedding from the villus tip with no compensatory increase in cellular proliferation (Figs. 1 and 2)^17^. Similar atrophy also occurs in enteroid cultures. As a result of cell loss by apoptosis with no accompanying change in cellular proliferation (Figs. 1 and 2), cells are shed into the enteroid lumen and these are not immediately replaced. Junctional complexes hold remaining epithelial cells together whilst there is likely pressure from the build-up of apoptotic bodies within enteroid lumens. This results in the enteroids gaining a more spherical morphology. We have quantified this enteroid ‘rounding’ in terms of circularity from 2-dimensional images and have validated this as a reproducible and robust method of cell death analysis in enteroids following cytokine treatment (materials and methods). Three days post passage, untreated enteroids had circularity values of ~0.35, however, circularity increased to ~0.65 48 h following 100 ng/mL TNF and ~0.9 with conditioned media from LPS-activated BMDCs in C57BL/6J proximal tissue-derived enteroids (Fig. 3a). We validated the reproducibility of the scoring method by assessment of intra-scorer variability where the same person (L.J.) scored images from the same 12 enteroids with a range of different circularities 1 week apart (Fig. 3b), and inter-scorer variability where two different blinded scorers (L.J. and C.D.) assessed enteroid circularity from the same 12 enteroid images (Fig. 3c). We also determined that there was no correlation between enteroid area and circularity as long as organoids were above a critical size at the start of each experiment, which equated to 3 days post passage or an initial baseline circularity value <0.4 (Fig. 3d).

To further validate the assessment of circularity as a reliable indicator of cytokine-induced cell death in proximal tissue-derived enteroids, we observed a good positive correlation (*R*^2^ = 0.6) between enteroid circularity and active caspase-3 immunohistochemical staining (Fig. 3e). TNF activates apoptosis via caspase-8 and active caspase-8 is observed at the villus tip during intestinal cell shedding^29^. Active caspase-8 was not detected at later time-points during enteroid studies (Supplementary Fig. 3). As enteroids derived from the middle or distal segments of the SI displayed a more rounded appearance in culture at baseline (Fig. 1a), they also showed a blunted response to TNF in terms of increased circularity compared with proximal tissue-derived enteroids (Fig. 3f). Future studies should therefore consider the use of the circularity method for determining enteroid response to injury, however this method is not suitable for comparing responses between enteroid regions. We therefore used proximal tissue-derived enteroids for the remainder of this study.

### Alternative NF-κB pathway signalling in small intestinal epithelial cells regulates the response to cytokine-induced injury

**Fig. 4:**
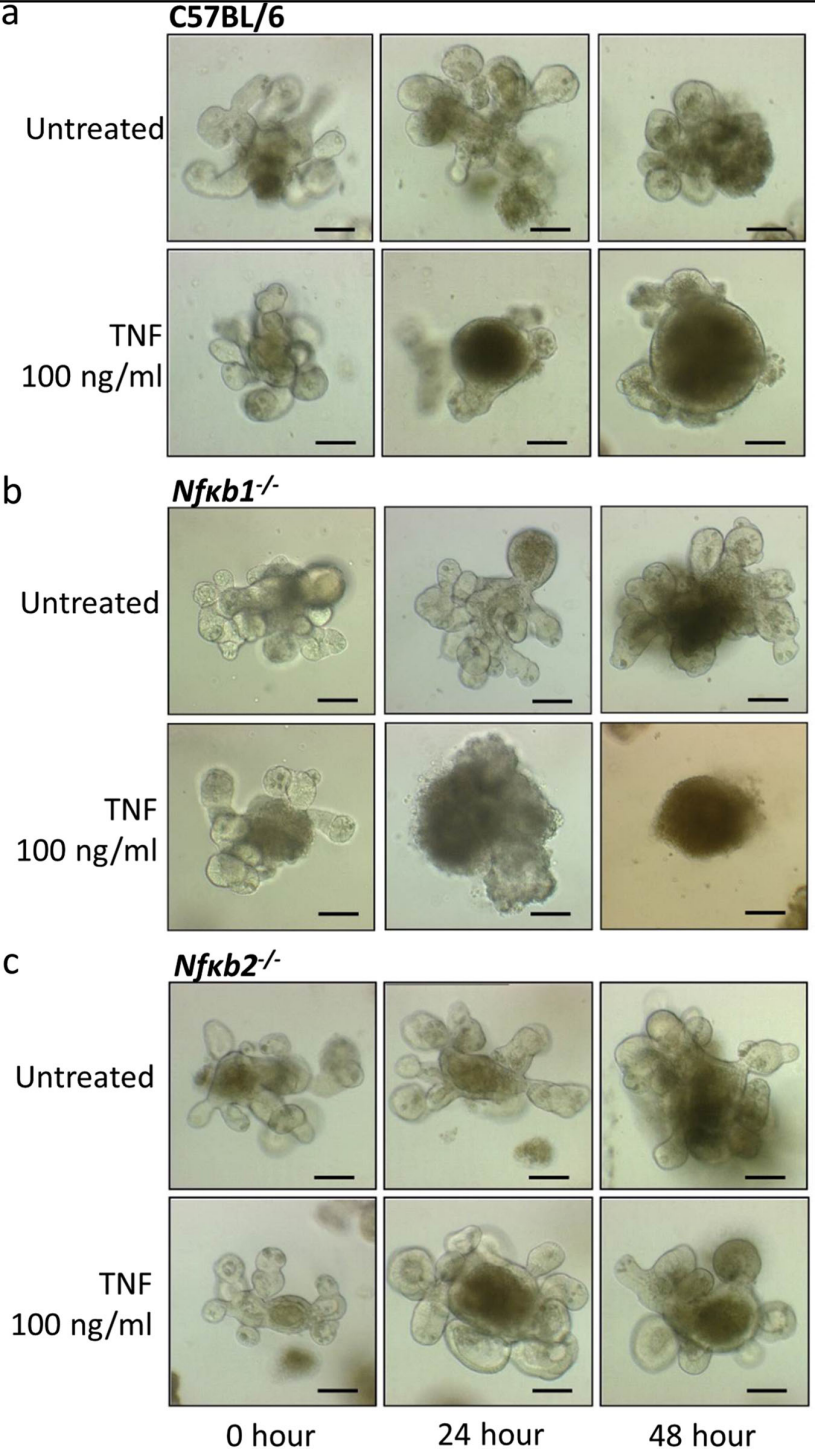
TNF induces circularity of C57BL/6 but not *Nfκb2*^*−/−*^ enteroids. TNF (100 ng/mL) induces enteroid rounding of C57BL/6J (**a**) and *Nfκb1*^*−/*−^ (**b**) proximal tissue-derived enteroids but not *Nfκb2*^*−/−*^ proximal tissue-derived enteroids (**c**) at 24 and 48 h time points following treatment. Scale bars = 100 µm.

**Fig. 5:**
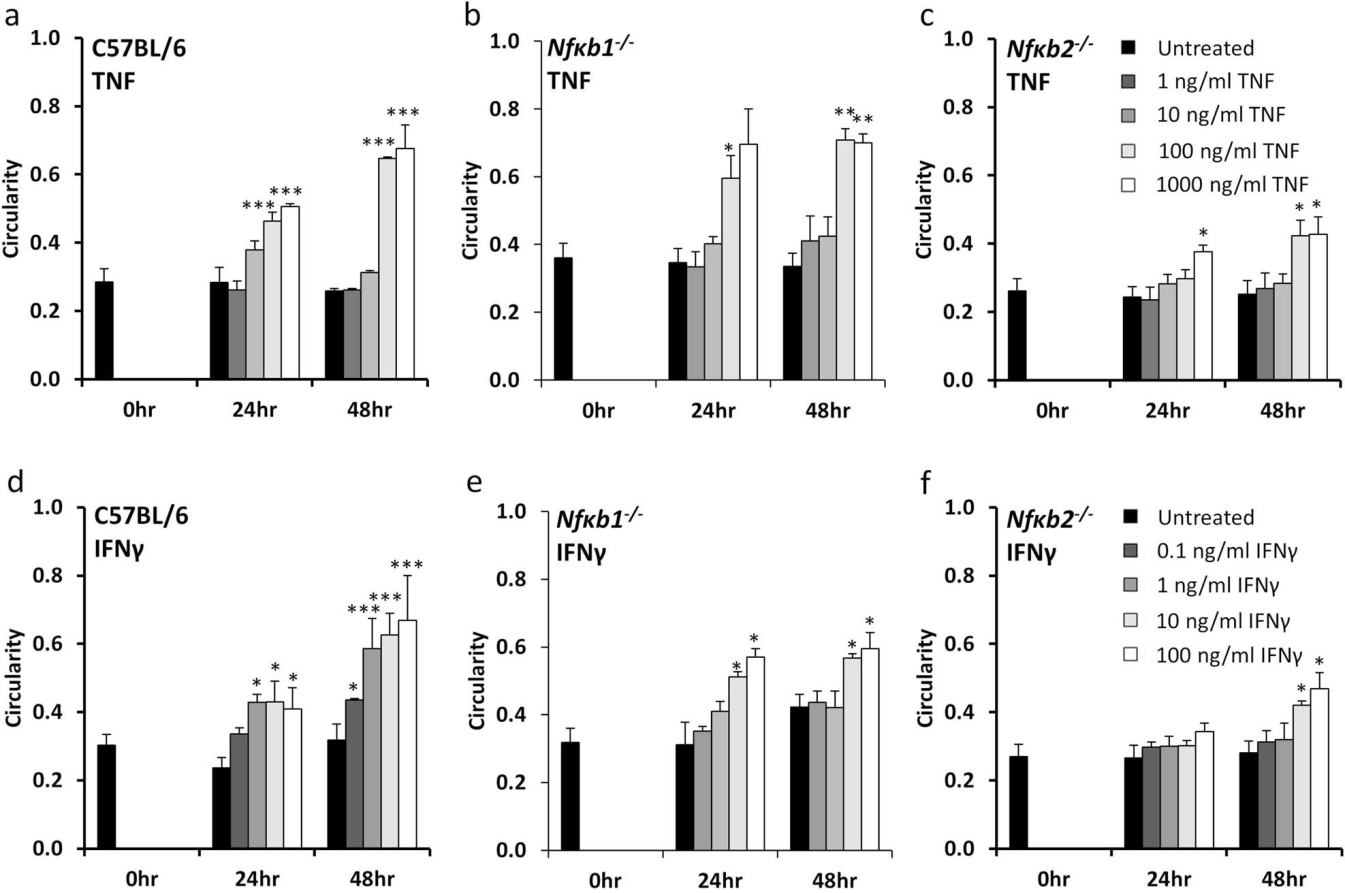
TNF and IFNγ-induced circularity is blunted in *Nfκb2*^*−/−*^ enteroids. Enteroid circularity is dose-dependently increased at 24 and 48 h time-points by treatment with TNF (**a**–**c**) and IFNγ (**d**–**f**) in C57BL/6J-derived enteroids (**a**, **d**) and *Nfκb1*^*−/*−^ enteroids (**b**, **e**), but is blunted in *Nfκb2*^*−/*−^ enteroids (**c**, **f**). *N* = 4, *n* = 6, *p < 0.05, **p < 0.01, **p < 0.001 compared with untreated of the same genotype at the same time point.

We have previously shown that *Nfκb2*^−/−^ mice are resistant to TNF-induced villus tip apoptosis and cell shedding compared with their wild-type C57BL/6J counterparts^17^. However, the mechanism responsible for this resistance could not be determined during these in vivo studies as it was impossible to quantify the contribution of specific cellular compartments such as epithelial cells or immune cells. Using proximal tissue-derived enteroids, we have now assessed the importance of NF-κB2 signalling specifically in the epithelial cell compartment in response to TNF and IFNγ stimulation. Both TNF and IFNγ caused C57BL/6J and *Nfκb1*^*−/−*^ mouse-derived enteroids to undergo morphological changes and round up over a 24-h period, with further noticeable rounding after 48 h (Fig. 4a, b). This response was however blunted in *Nfκb2*^*−/*−^ enteroids (Fig. 4c). A dose-dependent increase in circularity was observed in C57BL/6J and *Nfκb1*^*−/*−^ enteroids from 1 to 1000 ng/mL TNF and from 0.1 to 100 ng/mL IFNγ at both 24- and 48-h post treatment, however, no such increase was observed in *Nfκb2*^*−/*−^ enteroids (Fig. 5a–f) suggesting that alternative pathway NF-κB activation is an important modulator of the intestinal epithelial cell response to injury.

### LPS-activated bone marrow-derived dendritic cells induce enteroid rounding in a co-culture model and this is inhibited by epithelial NF-κB2 deletion

**Fig. 6:**
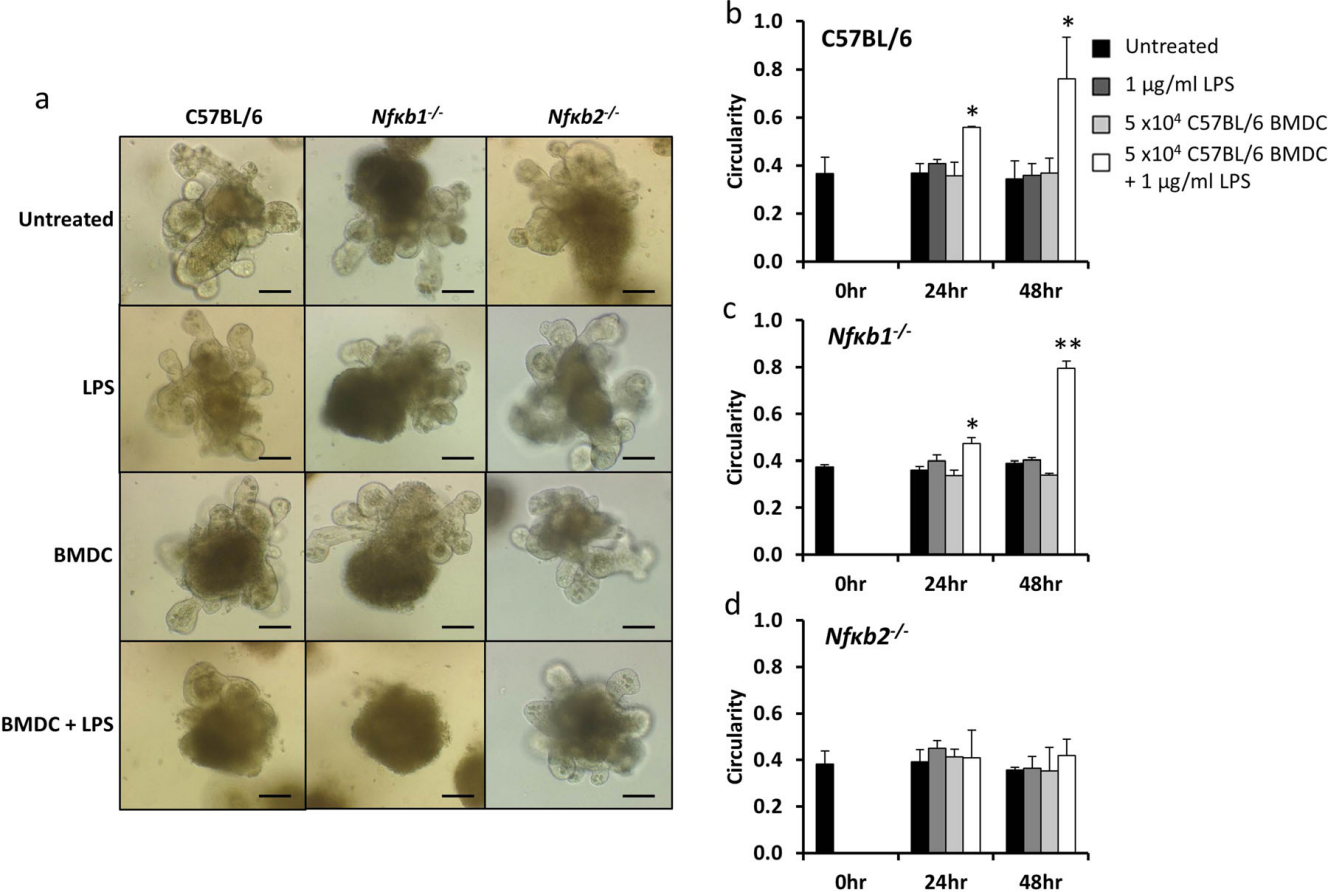
*Nfκb2*^*−/−*^ enteroids showed a blunted response to co-culture with LPS activated BMDCs. Enteroids were generated from the proximal SI of C57BL/6J, *Nfκb1*^*−/−*^ and *Nfκb2*^*−/−*^ mice and were either left untreated, treated with 1 µg/ml LPS, 5 × 10^4^ C57BL/6J-derived BMDC or 5 × 10^4^ C57BL/6J-derived BMDC plus 1 µg/ml LPS and subjected to light microscopy at 48 h (**a**). Circularity assessment was conducted at 0, 24 and 48 h time points for each stimulus in C57BL/6J (**b**), *Nfκb1*^*−/*−^ (**c**) and *Nfκb2*^−*/*−^ (**d**) enteroids. *N* = 3, *n* = 6, *p < 0.05, **p < 0.01 compared with untreated of the same enteroid genotype at the same time point. Scale bars = 100 µm.

LPS administration to mice in vivo resulted in abundant villus tip apoptosis and cell shedding 1.5 h following intraperitoneal injection. We have previously shown *Tnfr1*^−/−^ and *Tlr4*^*−/−*^ mice to be resistant to LPS-induced cell shedding, suggesting that LPS acts via TLR4 on immune cells to induce ligands that bind the TNFR1 on epithelial cells to initiate the shedding process^17^. Dendritic cells have important functions in the innate and adaptive immune systems and are activated by LPS. We therefore co-cultured C57BL/6J-derived BMDCs with C57BL/6J, *Nfκb1*^*−/*−^ or *Nfκb2*^*−/−*^ enteroids and tested BMDCs that had or had not been activated by LPS. The direct application of 1 μg/mL LPS or unstimulated BMDCs had no morphological effect on any enteroid genotype (Fig. 6a) and we did not observe any significant differences in enteroid circularity following treatment (Fig. 6b–d). Interestingly, co-culture of C57BL/6J and *Nfκb1*^−*/*−^ enteroids with LPS-activated BMDCs resulted in enteroid rounding (Fig. 6a) and a significant increase in enteroid circularity at both 24- and 48-h post treatment (Fig. 6b, c). However, *Nfκb2*^*−/*−^ enteroids were resistant to rounding and showed no significant increase in circularity in co-culture with LPS-activated BMDCs at 24 or 48 h (Fig. 6a, d). This suggests that NF-κB2 signalling specifically in intestinal epithelial cells is modulated by the interaction with dendritic cells.

### Secreted factors from LPS-activated bone marrow-derived dendritic cells are responsible for organoid injury

**Fig. 7:**
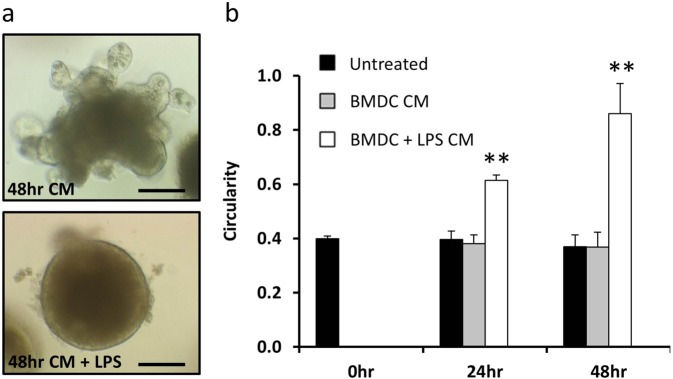
Conditioned media from LPS-activated BMDCs induced enteroid rounding. Enteroids derived from the proximal SI of C57BL/6J mice were untreated or treated with 50% conditioned media (CM) generated from 1 × 10^5^ BMDC/ml that were previously either untreated or treated with 1 µg/ml LPS for 48 h (**a**). Circularity assessment of enteroids was conducted at 0, 24 and 48 h time points for each stimulus (**b**). *N* = 3, *n* = 6, **p < 0.01 compared with untreated at the same time point. Scale bars = 100 µm.

We sought to determine whether the direct action of BMDC secreted factors on epithelial cells were responsible for the observed increase in C57BL/6J-derived enteroid circularity following stimulation with LPS. Conditioned media from unstimulated and LPS-stimulated BMDCs was placed onto C57BL/6J enteroids. Conditioned media from BMDCs did not induce an increase in enteroid circularity, however, conditioned media from LPS-activated BMDCs induced a significant increase in enteroid circularity at both 24- and 48-h post treatment (Fig. 7a, b). We assessed the cytokines that had been secreted by BMDCs into this conditioned media following stimulation with LPS and determined that several pro-inflammatory cytokines were upregulated. Initial analysis suggested that the amounts of IL-2, IL-4, IL-5 and IL-23 produced were minimal or below the lower limit of detection, hence these cytokines were not considered in subsequent experiments. TNF was upregulated in response to LPS and reached maximal concentrations of around 1 ng/mL (Table 1). However, this concentration was not high enough to stimulate an increase in enteroid circularity alone (Figs. 4 and 5). Significant increases in IL-6, IL-1β, IL-17A and IL-15 were also detected in the media of LPS-activated BMDCs in co-culture with enteroids and these cytokines may therefore have contributed to the observed increase in enteroid circularity. Secreted factors from dendritic cells are therefore able to modulate intestinal epithelial cell response to injury, which can be modelled in an enteroid system.

**Table 1:** Cytokines produced by C57BL/6J proximal tissue-derived enteroids, enteroids in co-culture with unstimulated BMDCs and enteroids in co-culture with LPS (1 µg/ml) activated BMDCs.

Cytokine	Enteroid (pg/ml)	Enteroid + BMDC (pg/ml)	Enteroid + BMDC + LPS (pg/ml)
IFN-γ	0.09 ± 0.02	0.05 ± 0.02	ND
IL-10	ND	98.42 ± 43.94	51.18 ± 30.89
IL-12 p70	ND	2.35 ± 2.03	320.85 ± 94.57
IL-15	0.40 ± 0.40	10.81 ± 6.37	186.74 ± 42.01*
IL-17A	0.01 ± 0.01	0.01 ± 0.01	1.51 ± 0.28*
IL-1β	ND	0.61 ± 0.28	42.90 ± 5.36*
IL-33	0.60 ± 0.15	0.30 ± 0.19	4.97 ± 2.92
IL-6	2.14 ± 0.82	31.61 ± 14.81	43967.35 ± 4220.72*
KC/GRO	4.16 ± 0.55	5.52 ± 1.96	103.43 ± 30.89
TNF	2.29 ± 0.51	180.37 ± 78.95	806.86 ± 335.23

Analysis from three independent experiments in duplicate, data presented ± SEM. ND = not detected. *p < 0.05 between enteroid + BMDC + LPS and enteroid + BMDC groups

## Discussion

Recent advances have enabled long-term three-dimensional culture of the mammalian SI epithelium^30–32^. However, many published studies have not considered the differences that exist in structure and function between different anatomical regions of the SI in vivo. The duodenum, jejunum and ileum are each specialised compartments which have distinct absorptive and secretory functions^26,33–36^. Previous studies in human and mouse enteroids have indicated that cultures retain their region-specific identities following multiple passage, in the absence of mesenchymal cell signalling or other niche factors^27^. For instance, the Gata 4 transcription factor is highly expressed in all epithelial cells of the duodenum, but shows greatly reduced expression further down the cephalocaudal axis, with no expression being detected in the terminal ileum^26,27^. Gata 4 has been shown to activate jejunal-specific genes such as *Clnd2* (encoding Claudin-2 protein) but represses ileal-specific genes such as *Slc10a2* (solute carrier family 10, member 2) and *Fabp6* (encoding fatty acid binding protein 6)^26,37^. Overexpression of Claudin-2 has been associated with inflammatory bowel disease and coeliac disease^38^ and is also upregulated in the intestine within 1 h of experimentally induced sepsis^39^. Regional differences in susceptibility occur for each of these pathologies and this is likely to result from differences in gene expression and environmental cues.

We therefore considered SI location when establishing enteroids and have identified variations in growth dynamics between three different regions of the intestine at baseline and following TNF treatment. These differences were also observed in parallel in vivo studies. In particular, Paneth cell numbers were increased from proximal to distal SI in vivo and in enteroid culture and we noted a greater amount of Paneth cell degranulation in the proximal SI both in vitro and in vivo in response to TNF. Previous studies have indicated that there is a greater degree of LPS-induced SI shedding and villus atrophy in the proximal SI compared with other regions^17^, however, loss of Paneth cell function within the crypt domains may also contribute to an alteration in the SI stem cell niche, thus perturbing cell production and dynamics further along the crypt-villus axis. We did not observe differences in the proliferative compartment along the length of the intestine by Ki67 immunohistochemistry (IHC), however, a large population of cells are labelled by Ki67 in both systems and more subtle differences in the proliferative compartment may be apparent by using more sensitive detection methods. It was difficult to compare the overall growth rate of organoids between the three regions, however, distal tissue-derived enteroids were more difficult to establish than those from other regions of the SI and displayed morphological differences that were maintained for greater than ten passages. Therefore, future studies should consider that following successive culture, proximal tissue-derived enteroids may outcompete distal tissue-derived derived enteroids in a mixed population.

One of the major cytokines that is responsible for the development of IBD and which is a major contributor to the cytokine storm and multiple organ dysfunction (including the gut epithelium) that occurs in sepsis is TNF^4,7,17^. The direct impact of TNF on enteroid morphology was striking. Over a period of 48 h, enteroids adopted a rounded appearance and many became almost spherical. This altered morphology was a likely result of increased apoptosis and cell shedding into the enteroid lumen. Analogous to bubble formation, a spherical appearance may result from the line of least tension across tight and adherens junctions with pressure build up from shed cellular debris and secretions within the enteroid lumen. The observed change in enteroid morphology was assessed by measuring circularity and this correlated linearly with active caspase-3 expression, thus validating the technique as a method for analysing cell death in this setting. This technique is much quicker than other similarly reported methods such as the assessment of branching morphogenesis by assessing the number and length of crypt buds^40^. The circularity assessment method is therefore useful for determining dose and time points for further investigations of a mechanistic nature, but requires optimisation and validation for each culture media formulation and damaging stimulus. The mechanisms of rounding are unlikely to be the same for all stimuli, however, this assay is particularly useful to determining the morphological impact on intestinal epithelia. Active caspase-3 is relatively stable compared with other caspases and has a half-life of 8–11 h^41^ and was detectable following TNF treatment for the entire time-course of our experiments. We also assessed the expression of active caspase-8 and whilst this was observed at the villus tip at early time-points as in previous studies^29^, this caspase is much less stable, cleaved components p43 having a half-life of 63 ± 5 min and p18 having a much shorter half-life of 7 ± 1 min^42^ and was not detected over the whole time-course of our experiments in enteroids, thus active caspase-8 activity did not positively correlate with enteroid circularity.

Upregulation of *NF-kB2* mRNA has been observed in colitis-associated dysplasia and carcinoma^43^. Our previous in vivo studies have also demonstrated that *Nfκb2*^*−/−*^ mice are less susceptible to the induction of experimental colitis, colitis-associated cancer and LPS-induced SI cell shedding^17,20^. However, the role of the intestinal epithelium in contributing to this protection was previously unclear as *Nfκb2*^*−/−*^ mice have a perturbed immune system that could have also contributed to the altered phenotype^44^. We have now demonstrated that *Nfκb2*^*−/−*^ proximal SI epithelium is resistant to TNF and IFNγ induced pathology in enteroids in the absence of contributions from the other cellular compartments that are present in vivo. This suggests that NF-κB2 signalling is an important determinant of intestinal epithelial cell homoeostasis. A range of concentrations of cytokines was tested in our experiments and alterations in circularity were noted at concentrations greater than 10 ng/ml TNF and 0.1 ng/ml IFNγ. These concentrations are higher than those detected in the circulation and tissues of human patients, however, faecal concentrations have been noted between 1 and 100 ng/g and are in line with the concentrations used in this study^11^. Murine TNF is a known regulator of both classical and alternative pathway NF-κB signalling and is likely to mediate its effects via TNFR1 and 2 ligation. However, it was surprising to also observe a decreased sensitivity to IFNγ in *Nfκb2*^−*/*−^ enteroids, though this is likely to be via an indirect mechanism resulting in the production of an NF-κB2 activator such as BAFF^45^. In addition to TNF and IFNγ, many cytokines contribute to the development of intestinal pathologies. We generated a co-culture model incorporating enteroids and BMDCs and showed that these cell populations could co-exist without any change to enteroid morphology. However, upon activation by LPS, BMDCs induced significant morphological changes in wild-type enteroids, whereas *Nfκb2*^*−/−*^ enteroids were seemingly protected. Cytokines generated in the model included TNF and IFNγ but both were produced at much lower concentrations than were required alone to induce circularity changes in enteroids and were similar to those observed in patients with pathology. This suggests that other secreted factors may also act synergistically with TNF and IFNγ to induce enteroid rounding in this setting. IL-6 was produced in abundance and could therefore contribute to modulating epithelial cell dynamics. An in-depth investigation of the BMDC secretome and assessment of cytokine combinations is now warranted to determine how the intestinal mucosal microenvironment regulates epithelial cell dynamics in combination with genetic susceptibility that may modulate the threshold for cytokines to induce a pathological response.

These data suggest that the NF-κB2 signalling pathway, specifically in intestinal epithelial cells, may be targetable to ameliorate intestinal and systemic inflammation.

## Materials and methods

### Animals

Ten- to 12-week old wild-type C57BL/6J (Charles River, Margate, UK) and *NfκB1*^*−/*−^ and *Nfκb2*^*−/−*^ mice^44,46^, bred on the C57BL/6J genetic background were maintained at the University of Liverpool under a 12:12 h light dark cycle and fed a standard pelleted diet. All mice were euthanized by cervical dislocation with ethical approval under UK Home Office legislation (Animals Scientific Procedures Act 1986) and local ethical approval.

### TNF treatment

Six female C57BL/6J mice were administered 0.33 mg/kg murine recombinant TNF (Peprotech Ltd., London, UK) by intraperitoneal injection and euthanized 1.5 h later. Six untreated female C57BL/6J were used as controls, mice were randomly allocated to two groups prior to treatment. SIs were excised, flushed with phosphate-buffered saline (PBS) pH 7.4 and fixed in 10% v/v neutral buffered formalin maintaining orientation using previously described methods^47^.

### Tissue preparation and scoring

Formalin-fixed SI was divided up into proximal, middle and distal thirds and bundled using methods previously described^47–49^. Bundles were routinely processed, embedded in paraffin wax in the transverse orientation and 4 µm thick tissue sections were stained with haematoxylin and eosin (H&E), direct red 80 (both from Sigma, Dorset, UK) or prepared for IHC.

For the quantification of apoptotic and proliferative intestinal epithelial cells in immunostained tissue sections, individual epithelial cells were counted from the base of the crypt to the mid-point of the villus tip in 20 well-orientated hemi crypt-hemi villi at 400× magnification from 6 mice per group (group sizes of 6 were estimated to be needed from previous studies to provide appropriate statistical power and the scorer was blinded to animal identifier at the time of analysis)^17^. For the quantification of apoptotic and proliferative intestinal epithelial cells in immunostained enteroid sections, individual cells were counted in the total circumference of 6 whole enteroids from at least 3 independent replicate studies at 400× magnification. Labelled cells were counted if in continuation with the epithelial monolayer. Intestinal epithelial cells were categorised according to the following criteria:

1. Normal if there was no or weak non-specific brown staining.
2. Apoptotic/mitotic if there was defined positive staining confined to cytoplasmic or nuclear borders.

### IHC and histochemical staining

Tissue and enteroid sections were deparaffinised and rehydrated. Standard histochemical techniques were used for H&E, direct red 80 (Sirius red) and alcian blue staining, the latter two being counterstained with haematoxylin to identify nuclei for quantification. Immunohistochemically stained slides were subjected to 1% v/v hydrogen peroxide in methanol to block endogenous peroxidases, followed by heat-mediated antigen retrieval in 0.01 M citrate acid buffer (pH 6) and then blocked with 1% w/v bovine serum albumin (Sigma, Dorset, UK). Rabbit polyclonal primary antibodies against active caspase-3 (AF835; R&D Systems, Abingdon, UK), Ki67 (AB15580; Abcam, Cambridge, UK) and olfactomedin-4 (#39141; Cell Signalling Technology, UK) were applied at 2.5, 2 and 0.25 μg/mL, respectively to sections for 2 h and visualisation was completed using an anti-rabbit Envision kit (Dako, Cambridge, UK) polymer followed by peroxidase substrate 3,3′-diaminobenzidine (DAB) (Sigma, Dorset, UK) using the manufacturer’s instructions. Sections were counterstained with haematoxylin.

### Bone marrow-derived dendritic cell extraction and culture

Femurs and tibias were harvested from male C57BL/6J wild-type mice and were mechanically cleaned to remove any surrounding tissues. Bones were transferred into 70% v/v ethanol for 2 min to sterilise their outsides. Bones were clipped at the top and bottom and a 24 G needle was used to flush out the bone marrow through a 40 μm cell sieve. Red blood cells were lysed, cells were quantified and resuspended in dendritic cell differentiation solution (RPMI containing GM-CSF (20 ng/ml), β-mercaptoethanol (50 μM), 10% w/v FCS, 1% w/v glutamine, 1% w/v penicillin/streptomycin; all from Sigma, Dorset, UK) in 10 cm petri dishes at a concentration of 2.5 × 10^5^/mL and plated at 20 mL/plate. Fresh media was added to dishes 4 days following and non-adherent BMDCs were used in experiments 7 days following isolation and differentiation.

BMDC conditioned media was generated by plating BMDCs in complete RPMI media as described above at concentrations of 1 × 10^3^, 1 × 10^4^, 1 × 10^5^ BMDC/mL. BMDCs were removed from culture media by centrifugation after 48 h and supernatants were immediately used in enteroid assays.

### Enteroid culture

Extraction and culture of SI crypts was carried out as previously described^32^ with modifications as indicated below. Male 10-week-old C57BL/6J mice were killed and the SI was rapidly removed and flushed with ice-cold PBS. The most proximal, middle and most distal 2 cm segments were isolated separately, opened out longitudinally, cut into 1 cm lengths and placed into PBS. Intestinal segments were washed 10 times in ice-cold PBS and incubated with ice-cold chelation buffer (20 mL of 2 mM EDTA in PBS) at 4 ^o^C with constant gentle agitation for 30 min. Chelation buffer was then replaced with 20 mL shaking buffer (43.3 mM sucrose, 59.4 mM sorbitol; Sigma, Dorset, UK) and shaken gently for 30 s to remove the majority of villi. Shaking buffer was replaced with fresh shaking buffer (20 mL) and crypts were then detached from the basal membrane by vigorous shaking by hand for 2 min. The crypt-enriched supernatant was filtered through a 70 μm cell strainer (BD Biosciences Heidelberg, Germany) and quantified using microscopy. The filtered crypts were centrifuged at 200 × *g* (4 °C, 10 min) and resuspended at 10,000 crypts/mL in Matrigel (BD Biosciences) containing growth factors; epidermal growth factor (50 ng/mL), R-spondin-1 (500 ng/mL) and Noggin (100 ng/mL) (R&D Systems). Approximately, 500 crypts in 50 μL Matrigel were seeded per well of a pre-warmed 24-well flat-bottomed plate and incubated at 37 °C for 10 min to polymerise the Matrigel. Finally, 500 μL of minigut culture medium was added to each well (advanced DMEM/F12, 1% l-glutamine, 1% Pen/Strep, 10 mM HEPES, 10 mM N2 supplement, 50 mM B27 supplement (Invitrogen)) and crypts were cultured at 37 °C in a 95% air, 5% CO_2_ atmosphere.

All enteroids were passaged at least once prior to use in an experiment. Passage was carried out weekly at a 1:4 split ratio. Media was removed and enteroids in Matrigel were resuspended in PBS and mechanically dissociated into crypts by passing through a 27 G needle. Dissociated enteroids were then centrifuged at 200 × *g* (4 ^o^C, 10 min), resuspended in fresh Matrigel containing growth factors and plated as above. Culture medium was changed and replaced with new media containing growth factors every 4 days.

### Enteroid treatments

C57BL/6J, *Nfκb1*^*−/−*^ and *NFκb2*^−*/−*^ enteroids (passage 2–12) were treated 3 days following passage by adding murine recombinant TNF (1–1000 ng/mL; PeproTech Ltd., London, UK), IFNγ (01–100 ng/mL; Peprotech Ltd.), C57BL/6J BMDCs (5 × 10^4^/well) with or without 1 μg/mL LPS diluted in 50% minigut media (containing 2× growth factors) and 50% complete BMDC media or conditioned media from BMDCs. High power images of 3 enteroids/well, conducted in duplicate were taken at 0, 24 and 48 h for circularity measurements and enteroids were harvested for histological analysis as described below.

### Enteroid histology

Following treatment with TNF, media was removed and replaced with 500 µL cell recovery solution (Corning, New York, USA) and agitated at 4 °C until the Matrigel was dispersed. Enteroids were then fixed in 2% v/v neutral buffered formalin. After 1-h fixation enteroids were centrifuged at 100×*g* for 1 min. The supernatant was discarded and 80–120 µl of pre-warmed Histogel^TM^ (Thermo Scientific, Massachusetts, USA) was mixed carefully with enteroids and transferred directly onto a section of Micropore^TM^ surgical tape forming a button when set. Sections of tape with Histogel were transferred into embedding cassettes and routinely processed and embedded in paraffin wax. Sections of Histogel containing enteroids (4 µm thick) were stained with H&E, direct red 80, or prepared for IHC as described above.

### Enteroid circularity assessment

Bright field microscopy was used to monitor enteroid development from crypt isolation and throughout subsequent passages and treatments. Images were taken using an Axiovert 25 microscope (Zeiss) and a 20× objective lens with a Hitachi HV-C20A camera. Enteroid morphology was assessed throughout experiments using 2D bright field images and a software-based formula for calculating circularity: 4*π* (area)/perimeter^2^. Two-dimensional images of enteroids were traced (three representative images per well conducted in duplicate) and analysis was conducted by recording enteroid area, perimeter and circularity using the analyse and measure function in ImageJ software^50^. Circularity values are possible between 0 and 1, with a score of 1 indicating a perfect circle. The circularity scoring method was adapted for enteroids from a previously reported system in mammary epithelial cultures^51^.

### Cytokine analysis

Media was removed from enteroids and enteroids in co-culture with BMDCs with and without LPS treatment 48 h post culture and centrifuged to remove debris. Samples were immediately frozen at −80 °C until further analysis and defrosted only once for further usage. Samples were run in duplicate and a multiplexing capable analysis platform selected for simultaneous cytokine profiling (QuickPlex SQ 120, Meso Scale Discovery, USA). The U-plex mouse cytokine assay system was employed for the analysis of a panel of markers as per manufacturer’s instructions. Cytokines analysed were: IFNγ, TNF, KC/GRO, IL-1β, IL-2, IL-4, IL-5, IL-6, IL-10, IL-12 p70, IL-15, IL-17, IL-23 and IL-33. Quantitative measurement of each marker was achieved using a four-parameter logistic regression method.

### Statistical analysis

Student’s *t* tests were carried out on normally distributed data within proximal, middle and distal regions of the SI and for cytokine production between LPS-treated BMDCs and untreated BMDCs. All additional normally distributed data were analysed by one-way ANOVA versus control group followed by Holm–Sidak post hoc analysis. Statistical tests that achieved *p* < 0.05 were considered significant.

## Supplementary information

Supplemental Figure legends

Supplemental Figure 1

Supplemental Figure 2

Supplemental Figure 3
