# Supplementary material for: NF-κB2 signalling in enteroids modulates enterocyte responses to secreted factors from bone marrow-derived dendritic cells
Source: Cell Death Dis. 2019 Nov 26;10(12):896. doi: 10.1038/s41419-019-2129-5 (PMC6879761; doi:10.1038/s41419-019-2129-5)
Supplement: Supplementary file 1 — Supplemental Figure legends [file 41419_2019_2129_MOESM1_ESM.docx]

**Supplemental Figure legends**

**Supplemental figure 1:** TNF does not modulate Olfm4 expression in small intestine at the time-points tested. Untreated and TNF treated proximal SI and enteroids stained for Olfm4 (a). Percentage of Olfm4 stained cells in untreated (white) and TNF treated (black) proximal, middle and distal small intestine (b). For animal study quantified from n=3 mice per group.

**Supplemental figure 2:** TNF does not reduce goblet cell number at the time-points tested. Proximal SI and enteroids stained with alcian blue (a). Percentage of alcian blue stained goblet cells in untreated (white) and TNF treated (black) proximal, middle and distal derived small intestine (top) and enteroids (bottom)(b). For animal study quantified from n=3 mice per group, for enteroid study n=6, N=3.

**Supplemental figure 3:** TNF activates caspase-8 in the small intestinal epithelium but is short lived. Proximal SI and enteroids stained for active caspase-8. Arrows indicate active caspase-8 positive cells with shedding morphology, and arrow heads indicate active caspase-8 positive cells without shedding morphology (a).
