# Supplementary figures and images for: NF-κB2 signalling in enteroids modulates enterocyte responses to secreted factors from bone marrow-derived dendritic cells
Source: Cell Death Dis. 2019 Nov 26;10(12):896. doi: 10.1038/s41419-019-2129-5 (PMC6879761; doi:10.1038/s41419-019-2129-5)

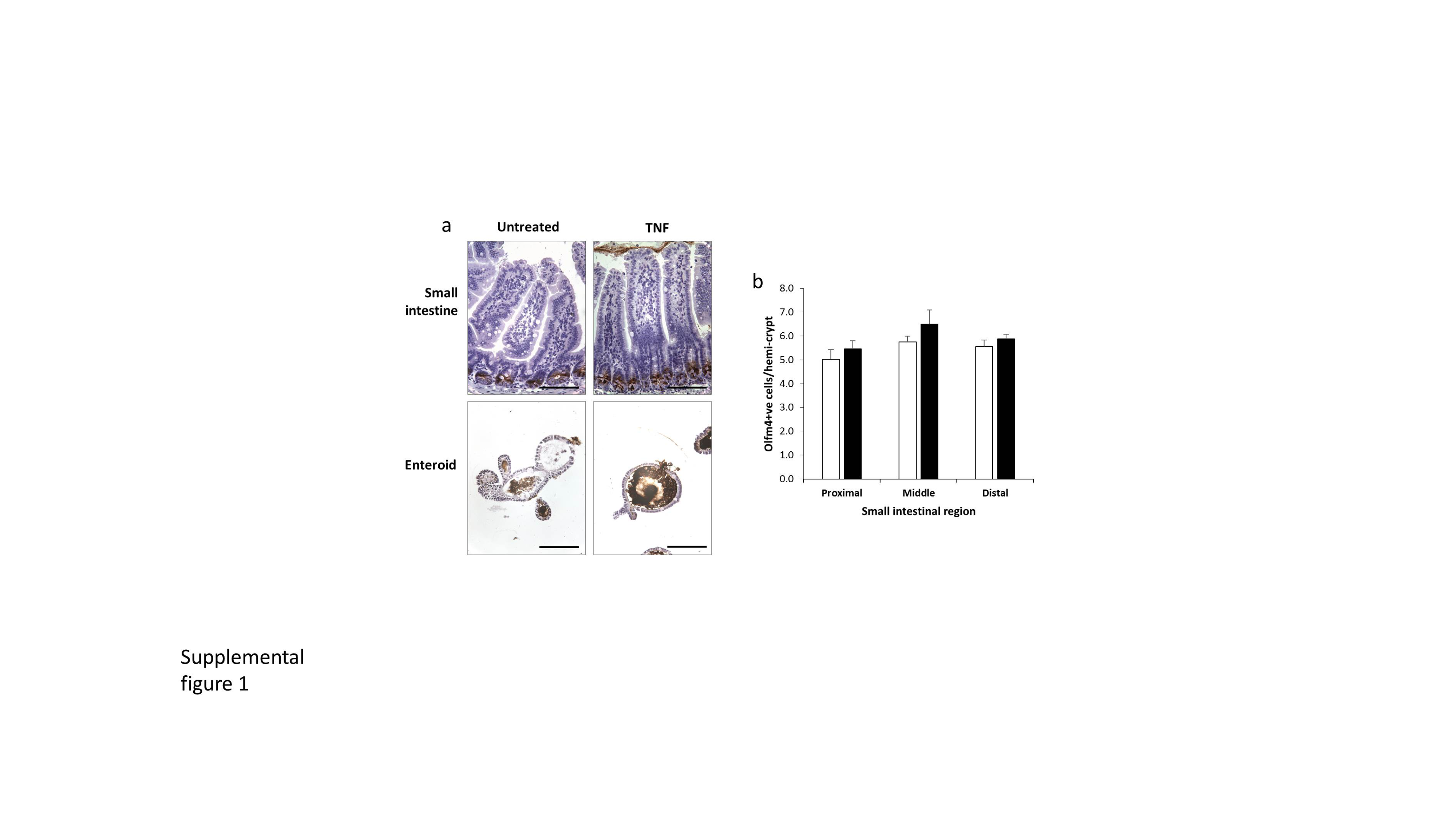

Supplement: Supplementary file 2 — Supplemental Figure 1 [file 41419_2019_2129_MOESM2_ESM.png]

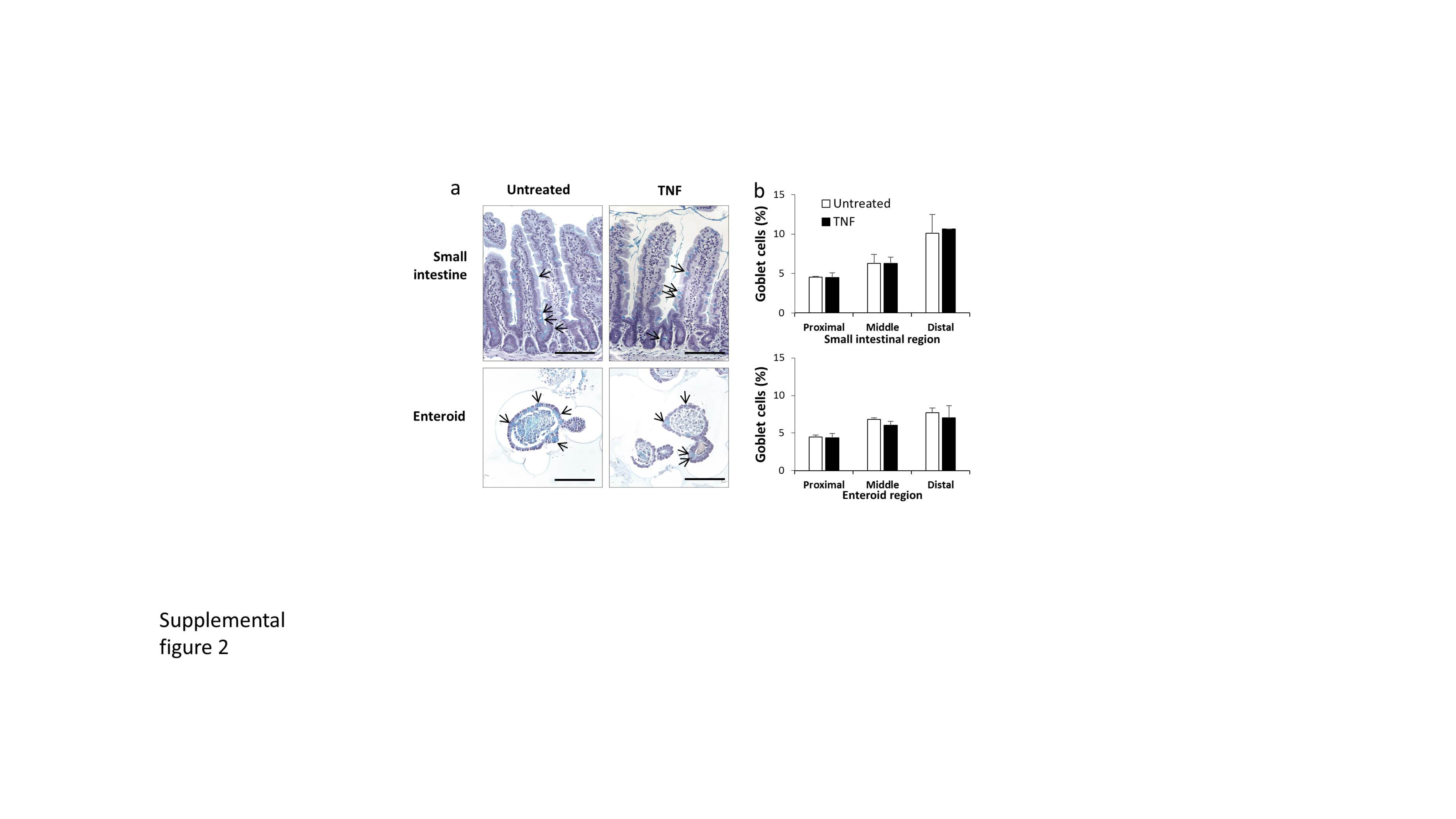

Supplement: Supplementary file 3 — Supplemental Figure 2 [file 41419_2019_2129_MOESM3_ESM.png]

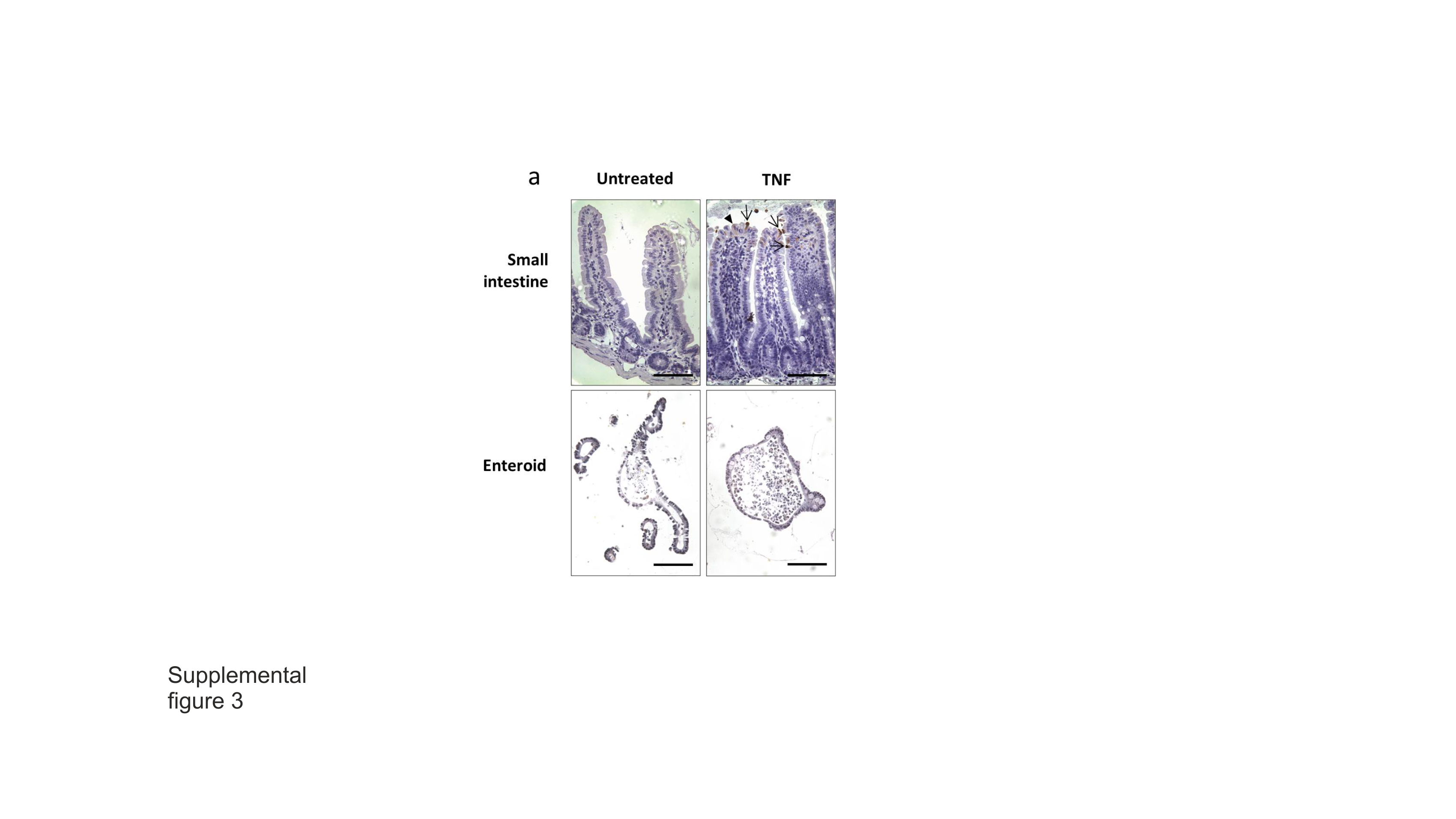

Supplement: Supplementary file 4 — Supplemental Figure 3 [file 41419_2019_2129_MOESM4_ESM.png]
